# Colorectal Cancer Characteristics Among Racial Minorities in the South Bronx: A 10-Year Retrospective Study at a Single Health Center

**DOI:** 10.7759/cureus.78667

**Published:** 2025-02-07

**Authors:** Haider Ghazanfar, Sameer Kandhi, Ahmed Alemam, Jasbir Makker, Bhavna Balar

**Affiliations:** 1 Internal Medicine, BronxCare Health System, Bronx, USA; 2 Gastroenterology and Hepatology, BronxCare Health System, Bronx, USA; 3 Gastroenterology, BronxCare Health System, Bronx, USA; 4 Medicine, BronxCare Health System, Bronx, USA

**Keywords:** african american, colon cancer screening, colorectal cancer, hispanics, colonoscopy

## Abstract

Background

Colon cancer is one of the leading causes of cancer-related deaths worldwide. The incidence of colorectal cancer has increased with time. Over the last several decades, people of color and racial minorities living in the South Bronx, the poorest congressional district in the United States, have suffered worse health outcomes than their White counterparts on virtually every health indicator.

Aim

To review the characteristics of patients belonging to racial and ethnic minorities diagnosed with colon cancer at the age of less than 50 and compare it to those who develop colorectal cancer more than or equal to the age of 50 years from a single health center in the South Bronx region.

Methods

We conducted a single-center, retrospective, observational study of patients who were followed from July 2011 to June 2021 at Bronx Care Health System. Patients aged 18 years and older who were diagnosed with colorectal cancer in our health system were included in the study. Patients younger than 18 years of age, who were pregnant, or who were diagnosed with colorectal cancer at other institutions were excluded.

Results

A total of 159 patients were included in the study. Gastrointestinal bleeding was the most common clinical presentation in 41 patients (25.8%), while iron deficiency anemia was the most common laboratory finding in 25 patients (15.7%). Sigmoid colon in 42 (26.4%) and ascending colon in 41 (25.8%) patients were the two most common locations of diagnosed colorectal cancers, and about 77 (48.4%) of the patients had stage 3 or 4 colorectal cancer at the time of diagnosis. The result of our study highlights the importance of getting a colonoscopy for colon cancer screening done at the age of 45 years. Sigmoid and ascending colon were found to be the two most common locations of colorectal cancer in our patient population. Most of the patients who present with colon cancer at an early age present at an advanced stage. Close to 63 of the total 159 (39.6%) patients who were diagnosed with colorectal cancer had missed or declined their initial screening colonoscopy when offered.

Conclusion

Colon cancer is a common but mostly preventable disease, especially in people who are within the recommended age for screening colonoscopies. While gastrointestinal bleeding and anemia are generally the most common symptoms, we have observed that younger patients with colorectal cancer often show signs of weight loss and are typically diagnosed at more advanced stages when they seek medical care.

## Introduction

Colorectal cancer was the third most diagnosed cancer in men and women in the United States in 2019. It is the second leading cause of cancer deaths, and it has accounted for 9.4% of all cancer deaths in 2020 [1]. A study using the GLOBOCAN 2012 (Global Cancer Observatory) database concluded that the incidence of colorectal cancer is expected to increase by 80% by the year 2035 [2]. The incidence of colorectal cancer was found to be 36 per 100,0000 as per United States cancer statistics from 2003 to 2019 [3]. The age-adjusted colon cancer rates of males in the Bronx from 2015 to 2017 were reported to be 44.6 per 100,000 as compared to 44 per 100,000 in New York. In females, the age-adjusted colon cancer rates in Bronx were 30.7 per 100,000 as compared to 33.8 per 100,000 in the New York State [4]. 

The incidence of colorectal cancer varies between different regions and geographical locations. Social, environmental, and genetic factors play a role in its incidence. The highest incidence rates were reported for Alaskan Natives (88.5 per 100,000) followed by American Indian people (46.0) and Black race (41.7) compared to slightly lower incidence rates in the White race (35.7). Similar racial and ethnic disparities were seen in the mortality trends [5]. The following risk factors have been found to be associated with colorectal cancer: increasing age, African American ethnicity, male gender, presence of hereditary colon cancer syndromes, family history of colon cancer in a first-degree relative, ulcerative colitis, Crohn’s disease, obesity, sedentary lifestyle, tobacco use, alcohol use, abdominopelvic radiation, consumption of a diet high in red and processed meat, history of renal transplant on immunosuppressive medication, diabetes mellitus, uretero-colic anastomosis, and coronary artery disease [6-9]. Residents of South Bronx were predominantly Hispanic people (64%) and the Black race (31%) along with other racial minorities like Asian and Filipino people [10]. The purpose of our study is to review the characteristics of patients belonging to racial and ethnic minorities diagnosed with colon cancer at the age of less than 50 and compare it to those who develop colorectal cancer more than or equal to the age of 50 years from a single health center in the South Bronx region.

## Materials and methods

We conducted a single-center, retrospective, observational study of patients who were followed from July 2011 to June 2021 at Bronxcare Health System. Patients aged 18 years and older belonging to racial minorities (Hispanic, Black, Filipino, and Asian patients) who were diagnosed with colorectal cancer in our health system and receiving follow-up care or treatment at our health center were included in the study (inclusion criteria). Patients younger than 18 years of age, who were pregnant, or who were diagnosed with colorectal cancer at other institutions were excluded.

All patients who met the inclusion criteria during that period were included in the study. We collected data about patients’ demographics, comorbid conditions, family history of colorectal cancer, clinical presentation, an indication of the colonoscopy, history of prior colonoscopies, history of missed colonoscopy, stage and location of colorectal cancer at the time of diagnosis, and cancer-related mortality.

The study was approved by the Institutional Board Review Committee at Bronx Care Health System. Data was entered in Excel sheets. Continuous variables were expressed as mean ± standard deviation. Categorical variables were entered as proportions. The data of the study was analyzed using the Statistical Package for the Social Sciences (SPSS), version 21.0 (IBM, Armonk, NY). Patients were divided into two groups based on their age; patients who were less than 50 years of age at the time of colorectal cancer diagnosis were placed in Group A, and patients who were equal to or older than 50 years of age were placed in Group B.

## Results

A total of 159 patients were included in the study. The mean age of the patients was 65.9 ± 12.6 years, and 82 (51.6%) patients among them were men. The majority of the patients were either Hispanic (88 (55.3%)) or African American (53 (33.3%)) patients. The mean body mass index (BMI) of the patients was 27.8 ± 7.3 kg/m^2^. Diabetes mellitus and coronary artery disease were the two most common comorbid conditions seen in our patients.

Patients had varied symptoms at the time of presentation, including gastrointestinal bleeding, weight loss, and abdominal pain. As noticed, gastrointestinal bleeding (occult/overt losses) was the most common clinical presentation (41 (25.8%)), while iron deficiency anemia was the most common laboratory finding in 25 patients (15.7%). The mean hemoglobin level at the time of diagnosis was 11.08 ± 2.49 g/dl.

About 28% of patients’ cancers were diagnosed in asymptomatic patients diagnosed during their colonoscopy performed either for screening or for surveillance purposes. About 15.1% were diagnosed during screening colonoscopy and 13.2% were diagnosed during surveillance colonoscopy.

Sigmoid colon in 42 (26.4%) and ascending colon in 41 (25.8%) patients were the two most common locations of diagnosed colorectal cancers, and about 77 (48.4%) of the patients had stage 3 or 4 colorectal cancer at the time of diagnosis. Sixty-three (39.6%) patients had either declined or missed their screening colonoscopy prior to their diagnosis. Cancer-related mortality was found in nine patients (5.7%). This is presented in Table 1.

**Table 1:** Patients’ demographics, comorbid conditions, family history of colorectal cancer, clinical presentation, history of prior colonoscopies, history of missed colonoscopy, stage and location of colorectal cancer at the time of diagnosis, and cancer-related mortality COPD, chronic obstructive pulmonary disease; CKD, chronic kidney disease; FOBT, fecal occult blood test; FIT, fecal immunochemical test.

Parameter	Result
Total cases	159
Age (mean ± SD)	65.9 ± 12.6
Gender (male)	82 (51.6%)
Race	
- African American	53 (33.3%)
- Hispanic	88 (55.3%)
- Others	18 (11.3%)
BMI (mean ± SD)	27.8 ± 7.3
- Underweight (<18.5)	6 (3.77%)
- Normal weight (18.5-24.9)	51 (32.07%)
- Overweight (25.0-29.9)	52 (32.70%)
- Class I obesity (30.0-34.9)	34 (21.38%)
- Class II obesity (35.0-39.9)	10 (6.28%)
- Class III obesity (≥40.0)	6 (3.77%)
Comorbidities	
- COPD	17 (10.7%)
- CKD	21 (13.2%)
- Diabetes mellitus	47 (29.6%)
- Coronary artery disease	30 (18.9%)
- Congestive heart failure	12 (7.5%)
Hemoglobin (mean ± SD)	11.08 ± 2.49
Clinical signs/symptoms	
- Abdominal pain	13 (8.2%)
- Abnormal exam (rectal mass)	1 (0.6%)
- Iron deficiency anemia	25 (15.7%)
- Weight loss	14 (8.8%)
- Abnormal CT of the abdomen	3 (1.9%)
- Altered bowel habits	9 (5.7%)
- Positive FOBT/FIT	8 (5.0%)
- Gastrointestinal bleeding	41 (25.8%)
- Screening colonoscopy	24 (15.1%)
- Surveillance colonoscopy	21 (13.2%)
Tumor location	
- Cecum	5 (3.2%)
- Ascending colon	41 (25.8%)
- Transverse colon	28 (17.6%)
- Descending colon	11 (6.9%)
- Sigmoid colon	42 (26.4%)
- Rectum	32 (20.1%)
Cancer stage	
- Stage IV	45 (28.3%)
- Stage III	32 (20.1%)
- Stage II	28 (17.6%)
- Stage I	34 (21.4%)
Cancer stage - Unknown stage	20 (12.6%)
Colonoscopy history - Missed/declined colonoscopy	63 (39.6%)
Cancer-related mortality	9 (5.7%)
Family history	
- First-degree relative	20 (12.6%)
- Second-degree relative	8 (5.0%)

Weight loss was found to be more common in patients in group A as compared to patients in group B, 3 (25%) vs 11 (7.5)% patients, respectively. Compared to no patients in group A, 28 patients (19.1%) in group B were diagnosed with stage 2 colorectal cancer. Ascending colon was the most common location of colorectal cancer in group A while sigmoid colon was the most common location in group B. Among the patients who were diagnosed with colon cancer at age less than 50 (group A), eight (66%) patients were diagnosed at stage 3 or 4. On the other hand, in group B consisting of patients aged 50 and above, 69 (47%) of patients were diagnosed with colon cancer at stage 3 or 4. This is presented in Table 2.

**Table 2:** Comparison of colorectal cancer diagnosed at age ≤50 and age >50 COPD, chronic obstructive pulmonary disease; CKD, chronic kidney disease; FOBT, fecal occult blood test; FIT, fecal immunochemical test.

Parameter	Colorectal cancer at age <50 (group A)	Colorectal cancer at age ≥50 (group B)
Total cases	12	147
Age (mean ± SD)	41.3 ± 6.6	67.9 ± 10.7
Gender (male)	9 (75%)	73 ( 49.7%)
Race		
- African American	4 (33.3%)	49 (33.3%)
- Hispanic	7 (58.3%)	81 (55.1%)
- Others	1 (8.3%)	17 (11.6%)
BMI (mean ± SD)	30.7 ± 6	27.6 ± 7.4
- Underweight (<18.5)	0 (0%)	6 (4.08%)
- Normal weight (18.5-24.9)	2 (16.66%)	49 (33.33%)
- Overweight (25.0-29.9)	3 (25%)	49 (33.33%)
- Class I obesity (30.0-34.9)	5 (41.66%)	29 (19.72%)
- Class II obesity (35.0-39.9)	2 (16.66%)	8 (5.44%)
- Class III obesity (≥40.0)	0 (0%)	0 (0%)
Comorbidities		
- COPD	0 (0%)	17 (11.6%)
- CKD	1 (8.3%)	20 (13.6%)
- Diabetes mellitus	2 (16.7%)	45 (30.6%)
- Coronary artery disease	1 (8.3%)	29 (20.4%)
- Congestive heart failure	1 (8.3%)	11 (7.5%)
Hemoglobin (mean ± SD)	11.14 ± 2.3	11.07 ± 2.5
Clinical signs/symptoms		
- Abdominal pain	2 (16.7%)	11 (7.5%)
- Abnormal exam (rectal mass)	0 (0%)	1 (0.7%)
- Iron deficiency anemia	2 (16.7%)	23 (15.6%)
- Weight loss	3 (25%)	11 (7.5%)
- Abnormal CT of the abdomen	0 (0%)	3 (2.1%)
- Altered bowel habits	1 (8.3%)	8 (5.4%)
- Positive FOBT/FIT	0 (0%)	8 (5.4%)
- Gastrointestinal bleeding	2 (16.7%)	39 (26.5%)
- Screening colonoscopy	2 (16.7%)	22 (15%)
- Surveillance colonoscopy	0 (0%)	21 (14.3%)
Tumor location		
- Cecum	0 (0%)	5 (3.4%)
- Ascending colon	5 (41.6%)	36 (24.5%)
- Transverse colon	0 (0%)	28 (19.1%)
- Descending colon	2 (16.7%)	9 (6.1%)
- Sigmoid colon	3 (25%)	39 (26.5%)
- Rectum	2 (16.7%)	30 (20.4%)
Cancer stage		
- Stage IV	5 (41.6%)	40 (27.2%)
- Stage III	3 (25%)	29 (19.7%)
- Stage II	0 (0%)	28 (19.1%)
- Stage I	2 (16.7%)	32 (21.8%)
Cancer stage - Unknown stage	2 (16.7%)	18 (12.2%)
Colonoscopy history - Missed/declined colonoscopy	3 (25%)	60 (40.8%)
Cancer-related mortality	0 (0%)	22 (15%)
Family history		
- First-degree relative	3 (25%)	17 (11.6%)
- Second-degree relative (SDR)	1 (8.3%)	7 (4.8%)

## Discussion

Recent studies have shown an increase in the incidence of colorectal cancer in patients under the age of 50 years [11]. A study analyzing the National Cancer Database (NCDB) found that 12.2% of the patients diagnosed with colorectal cancer were 50 years of age or younger at the time of diagnosis [12]. A study analyzing three Italian and Swiss case-control studies concluded that family history of colorectal cancer (odds ratio (OR) 4.50), ≥14 drinks/week of alcohol (OR 1.56), and a diet rich in processed meat (OR 1.56) were associated with increased risk of colon cancer in young adults [13]. Another study using the Florida Cancer Data System from 1981 through 2013 showed that patients younger than 50 years of age were significantly more likely to have advanced-stage colorectal cancer as compared to patients older than 50 [14]. In our study, eight (66%) out of the 12 patients younger than 50 years old had either stage 3 or 4 colorectal cancer at the time of diagnosis, compared to 79 (46.9%) out of the 147 patients older than 50 years of age. Seven (58.3%) out of 12 patients who had colon cancer at age less than 50 were younger than 45 years of age.

Obesity is a significant risk factor for colorectal cancer. Obesity and overweight have been shown to contribute to 11% of colorectal cancers in Europe [15]. A meta-analysis concluded that for every 5 kg/m^2^ increase in BMI, the associated risk of colon cancer increased by 30% in men and 12% in women [16]. BMI ≥30.0 kg/m^2^ has been associated with a higher risk of colorectal cancer in patients younger than 50 years of age [17]. Data from the Community Health Survey between 2002 and 2017 showed the highest percentage of adults (35%) who are obese (BMI of ≥30) and least percentage of adults who exercised in the past 30 days (70%) when compared to residents of other boroughs in the New York City [18]. In our study, the mean BMI for patients older than 50 years was 27.6 ± 7.4 kg/m^2^, while it was 30.7 ± 6 kg/m^2^ for patients below 50 years of age.

Patients with early-stage colon cancer are usually asymptomatic and are usually diagnosed during screening tests including screening colonoscopy. The majority of the patients however are diagnosed after they develop signs and symptoms of colorectal cancer [19]. Common signs and symptoms of colorectal cancer include abdominal pain, iron deficiency anemia, hematochezia, melena, weight loss, and changes in bowel habits. According to a retrospective cohort analysis, most symptomatic patients presented with at least one of the following symptoms: change in bowel habits, rectal bleeding, or abdominal pain [20]. In our study, weight loss was more commonly reported as a symptom or clinical presentation in colorectal patients younger than 50 years as compared to patients older than 50 years. A retrospective study found that 13% of the patients diagnosed with colorectal cancer aged 50 years or younger reported weight loss [19].

According to NYC (New York City) Health, residents of Manhattan had a timely colonoscopy rate of 78% while residents of the Bronx had a timely colonoscopy rate of 68% [21]. As per the Behavioral Risk Factor Surveillance system from 2019, males (69%) were found to have lower screening rates as compared to females (71%). The report also showed that patients with established healthcare providers had a screening rate of 73% as compared to 33% in patients without a regular healthcare provider. Also, patients with insurance (71.5%) were more likely to get health insurance than patients without insurance (51%) [22]. Several studies have identified a lack of knowledge about the disease, absence of regular healthcare provider, fear of experiencing pain during the procedure, inadequate insurance coverage, inadequate provider recommendations, lack of transportation services to attend appointments, language barriers with providers, and lack of family support as barriers to getting colorectal screening [23]. These factors are more prevalent among the racial and ethnic minority groups resulting in lower colon cancer screening rates in these communities. There is a need to increase awareness and support social workers in these communities to improve the colorectal screening rate.

The study's limitations include a relatively small sample size, especially among individuals with age less than 50 years. Further large cross-sectional studies with larger sample sizes are needed. Moreover, the study population is predominantly Hispanics and African American, reflecting our community's population characteristics. Further studies are needed to include patients of other minorities/ethnicities.

## Conclusions

Colon cancer is a common but mostly preventable disease, especially in people who are within the recommended age for screening colonoscopies. While gastrointestinal bleeding and anemia are generally the most common symptoms, we have observed that younger patients with colorectal cancer often show signs of weight loss and are typically diagnosed at more advanced stages when they seek medical care.

## References

[1] Xi Y, Xu P (2021). Global colorectal cancer burden in 2020 and projections to 2040. Transl Oncol.

[2] Douaiher J, Ravipati A, Grams B, Chowdhury S, Alatise O, Are C (2017). Colorectal cancer-global burden, trends, and geographical variations. J Surg Oncol.

[3] (2024). Colorectal Cancer Incidence, United States—2003−2019. Centers for Disease Control and Prevention. https://stacks.cdc.gov/view/cdc/126261/cdc_126261_DS1.pdf.

[4] NYCDOH NYCDOH (2024). Bronx Borough. Health Equity Report. http://www.health.ny.gov/statistics/community/minority/docs/mcd_reports_2021/bronx_county_bronx_borough.pdf.

[5] (2024). CRC News: May 30, 2024. American Cancer Society. https://nccrt.org/crc-news-march-1-2023/.

[6] Thanikachalam K, Khan G (2019). Colorectal cancer and nutrition. Nutrients.

[7] Nottage K, McFarlane J, Krasin MJ, Li C, Srivastava D, Robison LL, Hudson MM (2012). Secondary colorectal carcinoma after childhood cancer. J Clin Oncol.

[8] Jemal A, Siegel R, Xu J, Ward E (2010). Cancer statistics, 2010. CA Cancer J Clin.

[9] Botteri E, Iodice S, Bagnardi V, Raimondi S, Lowenfels AB, Maisonneuve P (2008). Smoking and colorectal cancer: A meta-analysis. JAMA.

[10] (2024). The South Bronx: An Economic Snapshot. https://www.osc.ny.gov/files/reports/osdc/pdf/report-13-2024.pdf.

[11] Bailey CE, Hu CY, You YN (2015). Increasing disparities in the age-related incidences of colon and rectal cancers in the United States, 1975-2010. JAMA Surg.

[12] Gabriel E, Attwood K, Al-Sukhni E, Erwin D, Boland P, Nurkin S (2018). Age-related rates of colorectal cancer and the factors associated with overall survival. J Gastrointest Oncol.

[13] Rosato V, Bosetti C, Levi F, Polesel J, Zucchetto A, Negri E, La Vecchia C (2013). Risk factors for young-onset colorectal cancer. Cancer Causes Control.

[14] Moore KJ, Sussman DA, Koru-Sengul T (2018). Age-specific risk factors for advanced stage colorectal cancer, 1981-2013. Prev Chronic Dis.

[15] Bardou M, Barkun AN, Martel M (2013). Obesity and colorectal cancer. Gut.

[16] Kyrgiou M, Kalliala I, Markozannes G (2017). Adiposity and cancer at major anatomical sites: Umbrella review of the literature. BMJ.

[17] Sanford NN, Giovannucci EL, Ahn C, Dee EC, Mahal BA (2020). Obesity and younger versus older onset colorectal cancer in the United States, 1998-2017. J Gastrointest Oncol.

[18] (2024). Bronx Community Health Dashboard: Colorectal Cancer. http://www.montefiore.org/documents/communityservices/OCPH-Dashboard-colorectal-cancer.pdf.

[19] Moreno CC, Mittal PK, Sullivan PS (2016). Colorectal cancer initial diagnosis: Screening colonoscopy, diagnostic colonoscopy, or emergent surgery, and tumor stage and size at initial presentation. Clin Colorectal Cancer.

[20] Thompson MR, O'Leary DP, Flashman K, Asiimwe A, Ellis BG, Senapati A (2017). Clinical assessment to determine the risk of bowel cancer using Symptoms, Age, Mass and Iron deficiency anaemia (SAMI). Br J Surg.

[21] (2024). NYC Vital Signs. Colorectal Cancer Prevention and Detection: Timely Screening among New Yorkers Ages 50 and Older. https://www.nyc.gov/assets/doh/downloads/pdf/survey/colorectal-cancer-screening-2020.pdf.

[22] (2024). Colorectal Cancer Screening in New York State. Progress Report 2019. Progress Report.

[23] (2024). Colorectal Cancer. Facts and Figures 2020-2022. American Cancer Society. https://www.cancer.org/content/dam/cancer-org/research/cancer-facts-and-statistics/colorectal-cancer-facts-and-figures/colorectal-cancer-facts-and-figures-2020-2022.pdf.

